# Asymptomatic congenital tracheal stenosis

**DOI:** 10.1002/rcr2.472

**Published:** 2019-08-01

**Authors:** Toshiyuki Sumi, Takumi Ikeda, Hisashi Nakata

**Affiliations:** ^1^ Department of Pulmonary Medicine Hakodate Goryokaku Hospital Hakodate Japan

**Keywords:** Complete tracheal rings, congenital tracheal stenosis, tracheal bronchus

## Abstract

Congenital tracheal stenosis (CTS) is often identified by characteristic wheezes and cyanosis in childhood. However, an asymptomatic progression to adulthood is rare. Asymptomatic latent CTS cases with a mild degree of stenosis may exist.

## Clinical Image

**Figure 1:**
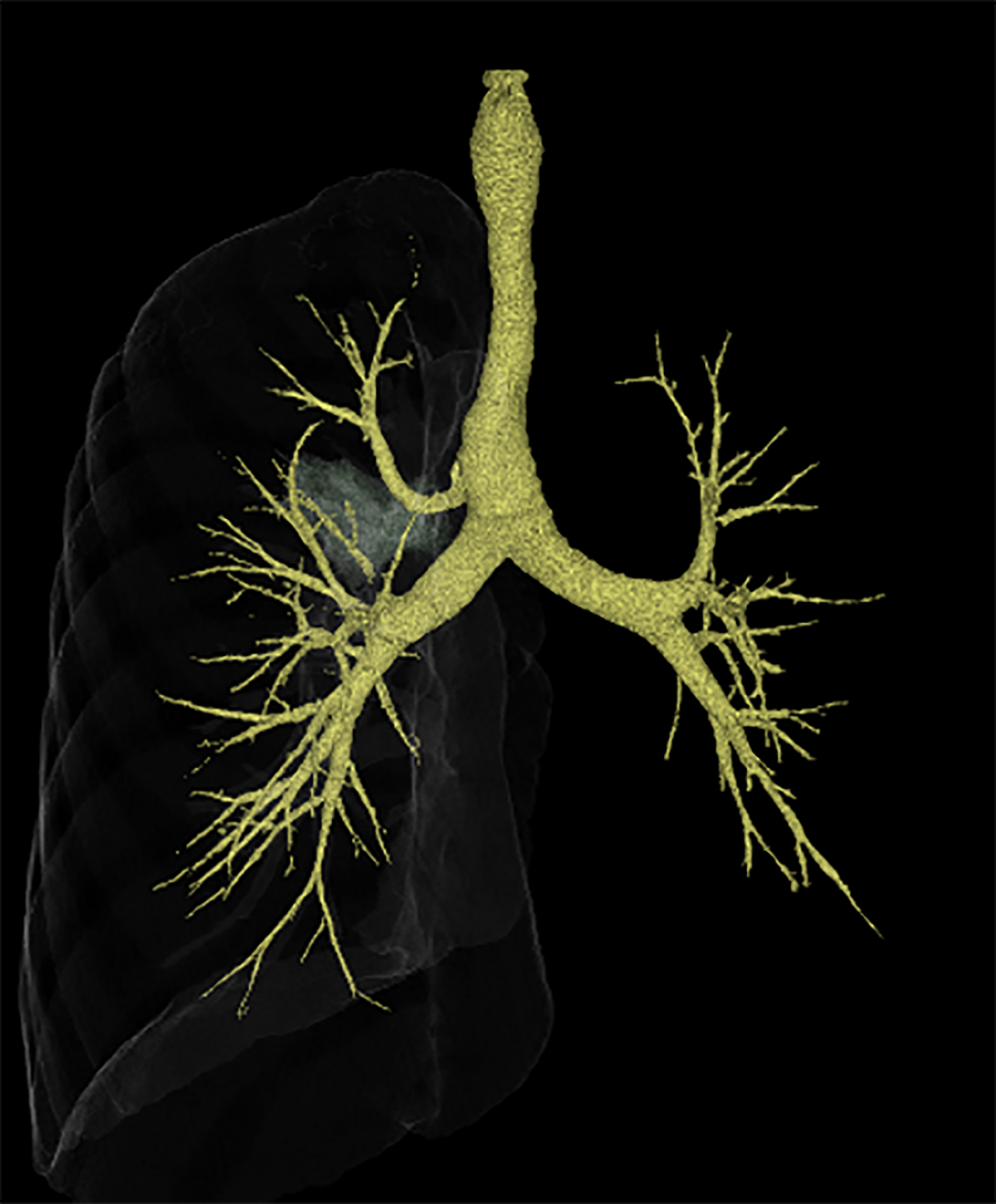
Computed tomography revealed a tracheal bronchus and a tracheal stenosis.

**Figure 2:**
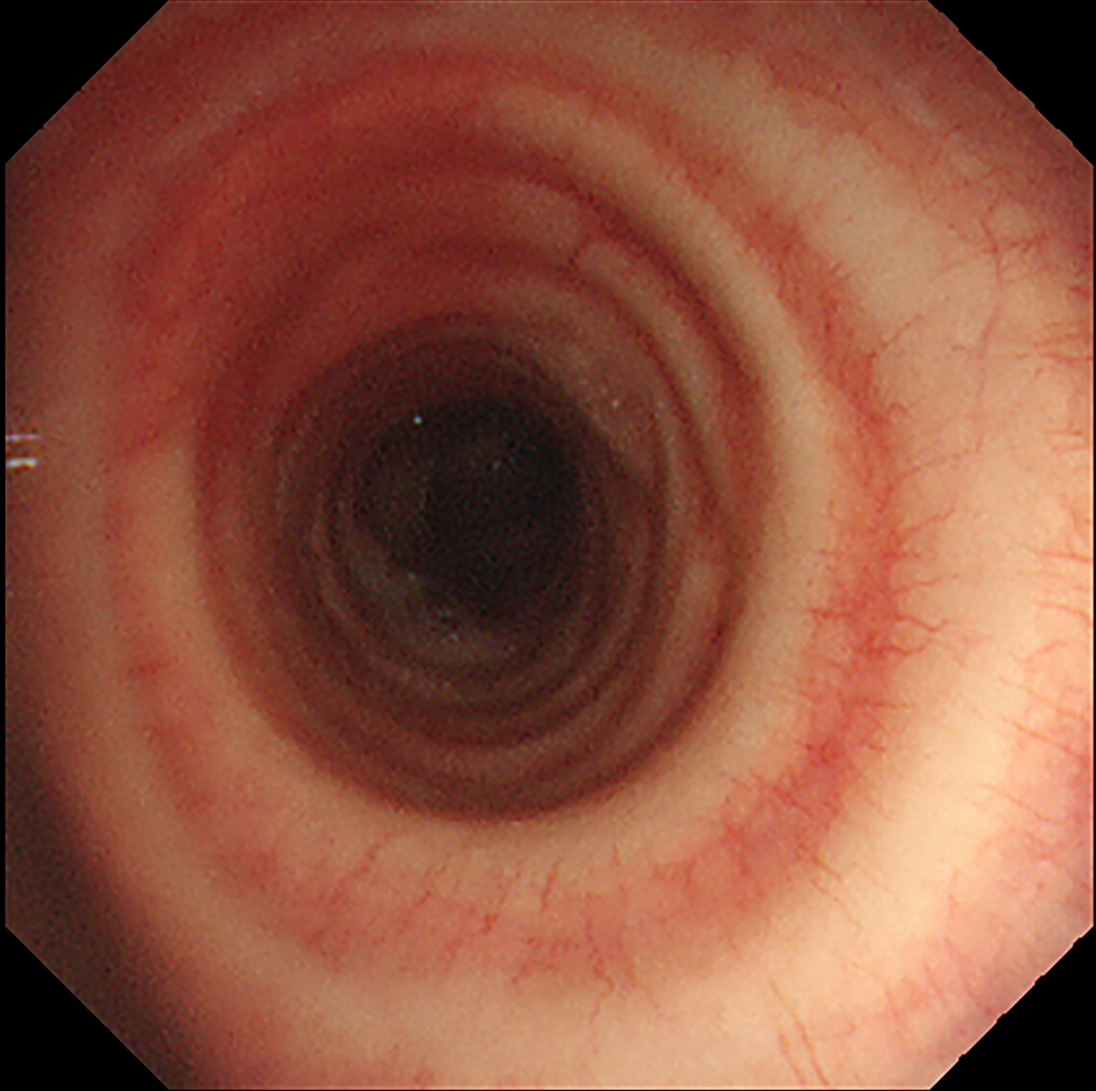
Bronchoscopy showed a defect in the tracheal membrane with the presence of concentric cartilage rings.

A 44‐year‐old man presented with an abnormality in chest radiograph at a medical checkup. Computed tomography revealed a tracheal bronchus and a mild degree of tracheal stenosis (Fig. 1). Bronchoscopy showed a defect in the tracheal membrane with the presence of concentric cartilage rings at the site of tracheal narrowing (complete tracheal rings) (Fig. 2). Based on these distinctive bronchoscopic findings, a diagnosis of congenital tracheal stenosis (CTS) was made. Abnormal bronchial branching patterns, such as a tracheal bronchus, are also common in CTS. The patient displayed no symptoms and was followed up without therapy.

CTS is often identified by characteristic wheezes and cyanosis in childhood. However, an asymptomatic progression to adulthood is rare. The adult trachea has an inner diameter of about 15–20 mm 1. Our patient displayed no exercise‐related symptoms until now because his tracheal inner diameter was 16 mm, with a mild degree of stenosis. Respiratory function test was also within normal limits. As described herein, asymptomatic latent CTS cases may exist.

### Disclosure Statement

Appropriate written informed consent was obtained for publication of this case report and accompanying images.

## References

[1] Randestad A , Lindholm CE , and Fabian P . 2000 Dimensions of the cricoid cartilage and the trachea. Laryngoscope 110:1957–1961. 11081618 10.1097/00005537-200011000-00036

